# The acute brain response to levodopa heralds dyskinesias in Parkinson disease

**DOI:** 10.1002/ana.24138

**Published:** 2014-05-28

**Authors:** Damian M. Herz, Brian N. Haagensen, Mark S. Christensen, Kristoffer H. Madsen, James B. Rowe, Annemette Løkkegaard, Hartwig R. Siebner

**Affiliations:** ^1^ Danish Research Center for Magnetic Resonance, Center for Functional and Diagnostic Imaging and Research, Copenhagen University Hospital HvidovreHvidovreDenmark; ^2^ Department of NeurologyCopenhagen University Hospital BispebjergCopenhagenDenmark; ^3^ Department of NutritionExerciseand SportsUniversity of CopenhagenCopenhagenDenmark; ^4^ Department of Neuroscience and PharmacologyUniversity of CopenhagenCopenhagenDenmark; ^5^ DTU InformaticsTechnical University of DenmarkLyngbyDenmark; ^6^ Department of Clinical NeurosciencesCambridge UniversityCambridgeUnited Kingdom; ^7^ Medical Research Council Cognition and Brain Sciences UnitCambridgeUnited Kingdom; ^8^ Behavioural and Clinical Neuroscience InstituteCambridgeUnited Kingdom; ^9^ Department of Clinical MedicineFaculty of Health and Medical SciencesUniversity of CopenhagenCopenhagenDenmark

## Abstract

**Objective:**

In Parkinson disease (PD), long‐term treatment with the dopamine precursor levodopa gradually induces involuntary “dyskinesia” movements. The neural mechanisms underlying the emergence of levodopa‐induced dyskinesias in vivo are still poorly understood. Here, we applied functional magnetic resonance imaging (fMRI) to map the emergence of peak‐of‐dose dyskinesias in patients with PD.

**Methods:**

Thirteen PD patients with dyskinesias and 13 PD patients without dyskinesias received 200mg fast‐acting oral levodopa following prolonged withdrawal from their normal dopaminergic medication. Immediately before and after levodopa intake, we performed fMRI, while patients produced a mouse click with the right or left hand or no action (No‐Go) contingent on 3 arbitrary cues. The scan was continued for 45 minutes after levodopa intake or until dyskinesias emerged.

**Results:**

During No‐Go trials, PD patients who would later develop dyskinesias showed an abnormal gradual increase of activity in the presupplementary motor area (preSMA) and the bilateral putamen. This hyperactivity emerged during the first 20 minutes after levodopa intake. At the individual level, the excessive No‐Go activity in the predyskinesia period predicted whether an individual patient would subsequently develop dyskinesias (*p* < 0.001) as well as severity of their day‐to‐day symptomatic dyskinesias (*p* < 0.001).

**Interpretation:**

PD patients with dyskinesias display an immediate hypersensitivity of preSMA and putamen to levodopa, which heralds the failure of neural networks to suppress involuntary dyskinetic movements. Ann Neurol 2014;75:829–836

L‐dopa is the most effective drug for the treatment of Parkinson disease (PD).^1^ However, long‐term L‐dopa treatment is complicated by the gradual development of involuntary movements referred to as L‐dopa–induced dyskinesias.^2^ Progressive neurodegeneration of dopaminergic neurons leads to increased metabolism of L‐dopa by nondopaminergic neurons, which can release dopamine into the striatal synaptic cleft, but lack a controlled reuptake mechanism.^4^ Alternatively, increased dopamine turnover has been proposed as a compensatory mechanism that allows preserved dopaminergic levels in early stages of PD.^5^ Both mechanisms result in nonphysiologic pulsatile stimulation of the putamen, which is thought to induce dyskinesias.

Recent studies have evidenced a substantial progress in understanding the cellular and molecular mechanisms underlying dyskinesias.^7^ Although these studies have shed important insights into the pathophysiology of dyskinesias, they tell little about how synaptic and cellular changes translate to in vivo dysfunction and behavior in patients affected by PD. Neuroimaging studies of dyskinesias in humans are sparse, because dyskinesias cause movement artifacts impairing data quality. In the current study, we adopted a novel strategy to bypass this problem. We performed functional magnetic resonance imaging (fMRI) in the time window between the intake of 200mg fast‐acting soluble L‐dopa and the onset of dyskinesias to avoid the presence of dyskinesias during fMRI.

A previous fMRI study has demonstrated abnormal neural activity in prefrontal areas in PD patients with dyskinesias after withdrawal of medication.^9^ However, it remains unclear how intake of L‐dopa modulates neural activity in patients with dyskinesias. Based on previous studies in animals^10^ and humans,^6^ we hypothesized that PD patients with dyskinesias would express abnormal dopaminergic modulation of the putamen and cortical areas. We further reasoned that this aberrant modulation of putaminal and cortical activity would emerge shortly after exposure to L‐dopa^13^ and predict the later manifestation of dyskinesias.

## Subjects and Methods

### Participants

We enrolled 36 patients fulfilling a clinical diagnosis of PD^15^ with predominant akinetic‐rigid symptoms. Patients had no dementia, major psychiatric illness, pacemaker, or any contraindication regarding MRI. They did not receive any sedatives or serotonergic medication in their current treatment.

Ten of the 36 patients were not able to undergo the MRI scan because they did not tolerate the withdrawal of dopaminergic medication (n = 8) or developed claustrophobia in the scanner (n = 2). Thirteen of the remaining 26 PD patients had clinically diagnosed choreiform peak‐of‐dose dyskinesias without off‐dyskinesias, or biphasic dyskinesias (L‐dopa–induced dyskinesia [LID] group), whereas the other 13 patients had no dyskinesias (No‐LID group). The presence and severity of dyskinesias were quantified using the Unified Dyskinesia Rating Scale (UDysRS). Dose of dopaminergic medication was higher in LID patients compared to No‐LID patients. However, the groups were closely matched with regard to disease duration and severity off and on medication. All clinical characteristics are listed in the Table. We also studied 13 age‐matched healthy individuals but without administration of L‐dopa. In accordance with the declaration of Helsinki, all participants gave their informed consent before entering the study, which was approved by the ethics committee of the Capital Region of Denmark (study No. H‐2‐2010‐146).

**Table 1:** Overview of Clinical and Demographic Characteristics

Variable	LID, n = 13	No‐LID, n = 13	Control, n = 13	*p*_uncorrected_
Gender	7 F	4 F	4 F	>0.5
Handedness	11 R	12 R	12 R	>0.5
Age, yr	68.9 ± 10.4	67.5 ± 6.5	68.4 ± 4.9	>0.1
Education, yr	14.9 ± 3.6	13.7 ± 3.4	15.8 ± 2.9	>0.1
MMSE	29.2 ± 1	29.6 ± 0.9	29.7 ± 0.6	>0.5
MoCA	28.3 ± 1.4	28.6 ± 1	28.9 ± 1.7	>0.5
BIS‐11	57.4 ± 7.5	55.5 ± 7.6	53 ± 6.6	>0.5
Disease duration, yr	7.5 ± 4.2	6.1 ± 3.3	—	>0.1
Medicine, LEDD	974.2 ± 415.9	672.3 ± 256.7	—	0.036^a^
Medicine, agonists	10	12		>0.5
UPDRS‐III‐OFF	32.5 ± 10.5	32.9 ± 6.8	—	>0.5
UPDRS‐III‐ON	20 ± 7.5	21.2 ± 5.1	—	>0.5
Δ UPDRS‐III	12.5 ± 4.9	11.6 ± 4	—	>0.5
UDysRS, objective	15.46 ± 8.7	—	—	—

Gender and handedness were compared using chi‐square tests. Chi‐square test was also used to compare whether dopamine agonists were more frequently added to L‐dopa therapy in LID (n = 10/13) compared to No‐LID patients (n = 12/13). Analysis of variance was used for comparison of age, education, MMSE, MoCA, and BIS‐11. Disease duration, LEDD, and UPDRS‐III were compared using independent samples *t* tests. Handedness was assessed using the Edinburgh Handedness Inventory.

a Indicates a significant difference in LEDD between the LID and No‐LID group. After dividing LEDD by body weight, the group difference remained significant (*p* = 0.002).

BIS‐11 = Barratt Impulsiveness Scale; F = female; LEDD = L‐dopa–equivalent daily dose; LID = L‐dopa–induced dyskinesia; MMSE = Mini‐Mental State Examination; MoCA = Montreal Cognitive Assessment; R = right; UDysRS = Unified Dyskinesia Rating Scale; UPDRS = Unified Parkinson Disease Rating Scale.

### Experimental Procedures

**Figure 1:**
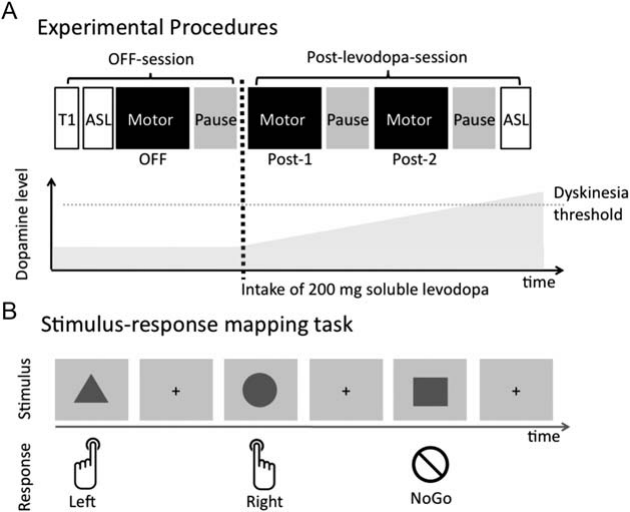
Experimental Procedures. (A) Timeline of experimental procedures. A first set of magnetic resonance imaging (MRI) scans was obtained after withdrawal of dopaminergic medication (OFF‐session). After initial structural and arterial spin labeling (ASL) scans, patients performed a motor task during functional MRI (fMRI; see below). The task‐related fMRI run lasted approximately 9 minutes, followed by a 5‐minute pause. Patients then received 200mg fast‐acting soluble oral L‐dopa, and the same sequence of fMRI scans was repeated twice after L‐dopa intake (post–L‐dopa session), followed by a second ASL scan. If dopaminergic levels reached the threshold for triggering dyskinesias, fMRI measurements were immediately discontinued. At least 1 post–L‐dopa fMRI scan after intake of L‐dopa could be acquired for all patients before emergence of dyskinesias. (B) Stimulus–response mapping task. The motor task consisted of 3 different stimuli indicating that participants should press a button with their left index finger or right index finger, or refrain from any motor response (No‐Go). Stimuli were presented for 750 milliseconds followed by a central fixation cross with a variable duration between 2,250 and 3,250 milliseconds, resulting in a mean inter‐trial interval of 3,500 milliseconds. Stimuli were pseudorandomly generated using PsychoPy (www.psychopy.org) with equal probability of each stimulus. Each session included 50 Left, 50 Right, and 50 No‐Go trials and lasted ∼9 minutes. Associations between stimulus and response were counterbalanced across participants and groups (L‐dopa–induced dyskinesia [LID], No‐LID, Control), but kept constant for each participant.

All PD patients underwent the same experimental procedures (Fig 1A). A first set of MRI measurements was acquired in a dopamine‐deprived state after withdrawal of all dopaminergic medication for a period corresponding to 6 half‐lives of the respective drug and at least 12 hours. In patients receiving dopamine agonists, this medication had to be stopped several days before the experiment. In these cases, we temporarily increased dosage of L‐dopa to maintain a similar level of L‐dopa–equivalent daily dose (LEDD) until 12 hours before the fMRI experiment. Once OFF‐scans were completed, patients received 200mg of fast‐acting soluble L‐dopa + 50mg benserazide (Madopar Quick; La Roche, Basel, Switzerland), and a second and third set of fMRI measurements were acquired after L‐dopa intake (post–L‐dopa scans). Approximately 10 minutes elapsed between application of L‐dopa and initiation of the first post–L‐dopa scan.

In each session, a motor task (see below), which lasted 9 minutes, was followed by a 5‐minute pause to avoid fatigue. A physician (D.M.H.) was always present inside the scanner room during MRI acquisition to visually observe whether dyskinesias emerged after L‐dopa intake. As soon as patients developed dyskinesias, MRI measurements were stopped.

Prior to the OFF‐fMRI scans, we acquired T1‐weighted structural brain scans. We additionally recorded arterial spin labeled (ASL) MRI to rule out putative differences in cerebral perfusion underlying L‐dopa–induced changes in the blood oxygenation level–dependent (BOLD) signal measured in fMRI.^16^ ASL scans were performed after acquisition of the T1‐weighted MRI and immediately after the post–L‐dopa fMRI scans (see Fig 1A). Healthy participants underwent the same set of MRI scans as PD patients, but no L‐dopa was given and the set of MRI measurements was only repeated twice. The rationale of including these healthy participants was to define a physiological measure of normal task performance and task‐related neural activity in the OFF session.

### Experimental Task

During fMRI, participants continuously performed a stimulus–response mapping task (see Fig 1B). Participants pressed the button of an MRI‐compatible computer mouse with the index finger of their right (Right condition) or left hand (Left condition), or refrained from any response (No‐Go condition), depending on arbitrary cues, which were presented pseudorandomly with equal probability.

Reaction times (RTs) and accuracy rates were analyzed to test for between‐group differences in task performance. Using RT as dependent variable, we computed a 3 × 3 × 2 analysis of variance (ANOVA) with the factors group (LID, No‐LID, control), run, and task laterality (left, right) after testing equality of distribution of error variance (*p* = 0.902, Levene test). Accuracy rates were compared using a nonparametric Kruskal–Wallis test, because Levene test showed that distribution of error variance was not equally distributed (*p* < 0.001). Between‐group differences in accuracy were assessed for the 3 conditions (Left, Right, No‐Go) and sessions. All group data are given in mean ± standard deviation (SD), and the significance threshold was set to *p* < 0.05 after Bonferroni correction.

### MRI

We used a 3T Verio scanner (Siemens, Erlangen, Germany) with a 32‐channel head coil. MRI data were preprocessed and analyzed using statistical parametric mapping software (SPM8.4667; Wellcome Trust Centre for Neuroimaging, London, UK).

A T1‐weighted structural image of the brain was acquired using a magnetization prepared rapid acquisition gradient echo sequence (field of view = 230mm, slice thickness = 0.9mm, repetition time [TR] = 1,900 milliseconds, echo time [TE] = 2.32 milliseconds, flip angle = 9°). T1‐weighted images were segmented to create individual gray matter, white matter, and cerebrospinal fluid masks for ASL analysis. ASL was used to map regional cerebral perfusion before and after L‐dopa intake.^16^ ASL involved FAIR‐Q2TIPS^17^ sequences with 3D‐GRASE^18^ single‐shot readout with background suppression (TR = 3,000 milliseconds, TE = 12.6 milliseconds, inversion time [TI] = 200, 400, 600, 800, 1,000, 1,200, 1,400, 1,600, 1,800, 2,000, 2,200, and 2,400 milliseconds, 1 average per TI, voxel size = 3.6 × 3.6 × 3.0mm^3^, vessel suppression with bipolar gradients, b = 10 s/mm^2^, 36 slices, whole brain coverage). We used Fast ASL and BOLD Bayesian Estimation Routine (FABBER) to analyze ASL‐based perfusion measurements.^19^

Task‐related BOLD signal changes were mapped using an echo planar imaging sequence (TR = 1,850 milliseconds, TE = 26 milliseconds, flip angle = 75°). A single fMRI volume consisted of 36 slices covering the entire brain (field of view = 192mm, slice thickness = 3.5mm, slice spacing = 0.2mm). Preprocessing comprised realignment, normalization, and spatial smoothing (full width at half‐maximum = 8mm), and high‐pass filtering (1/128Hz).

### Univariate Statistical Analysis of fMRI data

Analysis of task‐related BOLD signal changes was performed using the general linear model. The first‐level model specified 6 regressors of interest for each session, comprising Right, Left, and No‐Go and their first‐order temporal derivative. The influence of head movement artifacts was modeled by including 24 nuisance regressors derived from realignment.^20^ We also included recordings of respiration and cardiac pulsation as nuisance covariates.^21^

We performed independent samples *t* tests to assess between‐group differences in task‐related activity at the group level. Separate *t* tests were computed for Right, Left, and No‐Go after withdrawal of medication (OFF‐scan) to test for differences in task‐related activation at baseline.

We expected a gradual change in task‐related neural activity following L‐dopa intake. Therefore, using the contrast maps reflecting the temporal derivative of each regressor (Left, Right, NoGo), we compared L‐dopa–induced linear changes in task‐related activity in LID and No‐LID patients. Here, we included the first post–L‐dopa scan, which was available for all patients (see Results). This test enabled us to identify rapidly emerging changes in task‐related activity before the clinical manifestation of dyskinesias.

All fMRI results were thresholded at a cluster‐corrected threshold of *p* < 0.05 using the familywise error correction method. Given our a priori hypothesis that PD^22^ and dyskinesias^23^ would be associated with abnormal activity of the putamen, we defined the bilateral dorsocaudal putamen as a region of interest (ROI) and applied small volume correction (SVC) using a sphere 10mm in radius on the Montreal Neurologic Institute coordinates ±28, 2, 2 (x, y, z).^24^

### Post Hoc Analyses

Using fMRI data from the first post–L‐dopa scan, we performed a post hoc regression analysis to assess whether the gradually increasing neural response to L‐dopa correlated with severity of the day‐to‐day symptomatic dyskinesias as reflected by the objective UDysRS scores. In each patient, we extracted the first eigenvariate of regions, which displayed abnormal modulation by L‐dopa in the LID group (ie, the presupplementary motor area [preSMA] and bilateral putamen, see Results). We then entered the individual values into a linear regression model with severity of dyskinesias as dependent variable using SPSS v20 (IBM, Armonk, NY). The same regression analysis was repeated using the individual Unified Parkinson Disease Rating Scale (UPDRS)‐III as dependent variable. Results were thresholded at *p* < 0.05 after Bonferroni correction.

We used the same regional eigenvariates as in the regression analysis to predict whether an individual PD patient was diagnosed with LID. We entered the parameters of each region (n = 3) and patient (n = 26) as predictors in a binary classifier to see whether the parameters could classify a given patient as dyskinetic or nondyskinetic. For classification analysis, we applied a linear support vector machine (SVM; c‐value = 1)^25^ implemented in LIBSVM v3.17 as described previously.^26^ We used leave‐one‐out cross‐validation to assess classification accuracy, the true‐positive rate (sensitivity), and false‐positive rate (1 − specificity) and permutation tests (10,000 permutations) to derive the corresponding probability value.

Additional post hoc analyses included voxelwise comparisons of the mean BOLD signal between groups to test whether differences in task‐related BOLD signal changes could be explained by regional reduction in mean BOLD signal, for instance due to regional iron accumulation.^27^ Because L‐dopa might induce regional changes in cerebral perfusion, we submitted the ASL data to an ANOVA to test for putative interactions between group (LID and No‐LID) and state of medication (OFF and post–L‐dopa). These post hoc analyses had only one purpose, namely to exclude that between‐group differences in task‐related BOLD activity were associated with regional differences in mean BOLD signal or regional perfusion. Therefore, we applied SVC using spherical ROIs with 10mm radius. ROIs were centered on the peaks of the activation differences as revealed by fMRI.

## Results

### Dyskinetic Effect of L‐Dopa

The intake of 200mg L‐dopa provoked mild to severe peak‐of‐dose dyskinesias in 10 of 13 LID patients. Dyskinesias first developed in the foot on the side that was most affected by PD. Four LID patients developed dyskinesias after the first post–L‐dopa scan ∼20 minutes after L‐dopa intake. Patients with severe dyskinesias developed dyskinesias more rapidly than patients with mild dyskinesias (rho = −0.692, *p* = 0.009, Pearson correlation). There was no correlation between dyskinesia severity and LEDD (*p* > 0.1). Three of the 13 LID patients did not develop dyskinesias during the fMRI experiment, but showed mild dyskinesias after the fMRI experiment. These patients had the lowest objective UDysRS scores (6 points) in the LID group and received average levels of LEDD (mean = 883mg). None of the patients in the No‐LID group developed dyskinesias.

### Task Performance

Mean RT during the task was 667 ± 72 (SD) milliseconds in the control group, 684 ± 92 milliseconds in the No‐LID group, and 673 ± 103 milliseconds in the LID group. Error rates were <4% in all 3 groups and fMRI runs regardless of response condition. There was no difference in RT between group (*p* = 0.352), session (*p* = 0.204), or response condition (*p* = 0.222), and no significant interactions among the 3 factors. Likewise, we found no differences in accuracy rates between groups in any task or session (lowest *p* = 0.33).

### Task‐Related Activity in the OFF‐Medication State

**Figure 2:**
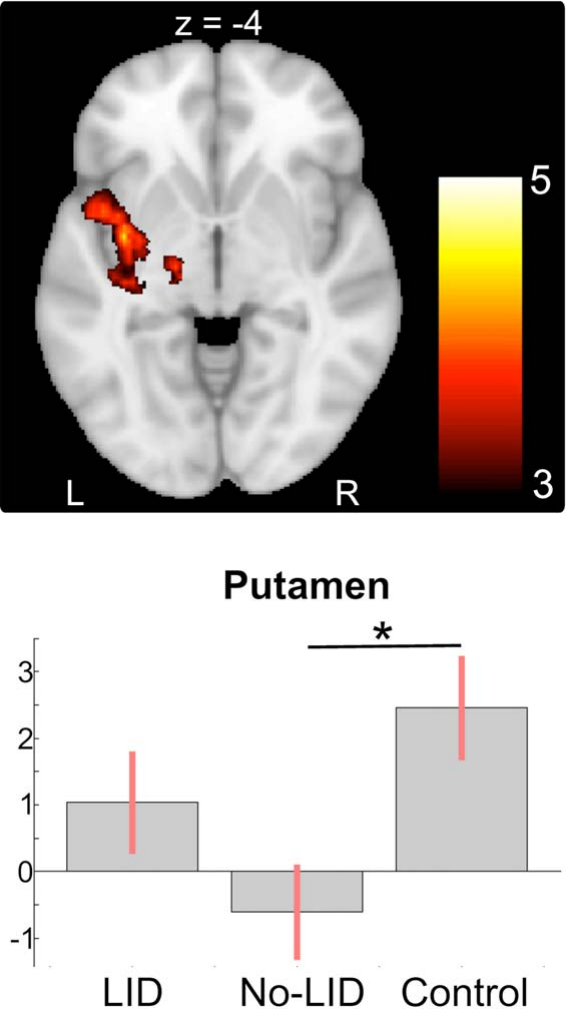
Differences in neural activation between groups in the OFF session. Parkinson disease patients without dyskinesias showed decreased activation in the left posterior putamen compared to healthy controls during right button presses. The effect sizes of the mean blood oxygenation level–dependent signal change are shown in the lower panel, demonstrating that magnitude of activation in L‐dopa–induced dyskinesia (LID) patients was in‐between patients without dyskinesias and healthy controls. L = left; R = right. The asterisk indicates a statistical difference of the mean at P < 0.05.

PD patients without dyskinesias showed decreased activation of the left posterior putamen during right button presses relative to healthy controls (peak at x, y, z = −34, −2, −4; *Z*_max_ = 3.4), whereas the magnitude of activation in LID patients was in‐between these two groups (Fig 2). This difference is unlikely to be caused by differences in lateralization of PD symptoms, with 5 of 13 LID patients and 7 of 13 No‐LID patients presenting with right‐lateralized symptoms. The mean BOLD signal intensity in the left putamen did not differ among groups. For left button presses and the No‐Go condition, there were no significant differences in task‐related activity between groups in the OFF session.

### Modulation of Task‐Related Neural Activity by L‐Dopa

**Figure 3:**
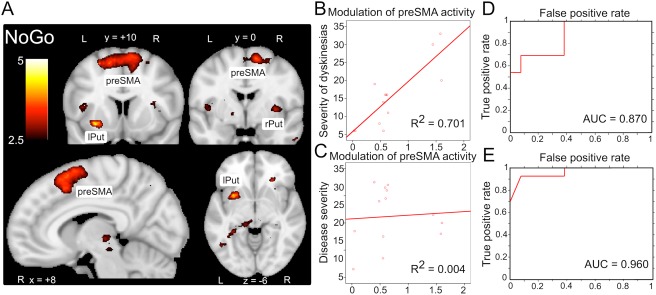
Abnormal modulation of neural activity following L‐dopa intake in L‐dopa–induced dyskinesia (LID) patients. (A) Analysis of time modulation of No‐Go after L‐dopa intake (first post–L‐dopa scan) showed a significantly stronger increase in activation of presupplementary motor area (preSMA) and bilateral putamen in LID patients compared to patients without dyskinesias. This was not observed during right or left button presses. Activations are shown in coronal, sagittal, and axial orientation. L = left; lPut = left putamen; R = right; rPut = right putamen. (B) Regression analysis showed that dopaminergic modulation of preSMA activity during No‐Go was a strong predictor of severity of emerging dyskinesia (*R*^2^ = 0.701, *p* < 0.001). (C) Dopaminergic modulation of preSMA activity did not predict severity of Parkinson symptoms (Unified Parkinson Disease Rating Scale‐III scores; *p* = 0.574). (D) The linear classifier significantly predicted whether an individual Parkinson disease patient had a diagnose of LID (accuracy = 80.8%, sensitivity = 69.2%, specificity = 92.3%, area under the curve [AUC] = 0.87, *p* < 0.001). (E) Three of 13 LID patients did not develop dyskinesias during the scan. Repeating the classifier for the LID patients who developed dyskinesias during the scan yielded 90% sensitivity and 92.3% specificity (AUC = 0.96, *p* < 0.001).

The first post–L‐dopa fMRI scan yielded differences in the responsiveness to L‐dopa between LID and No‐LID patients (Fig 3A). Compared to the No‐LID group, the LID group displayed an increased linear enhancement of task‐related activity in the preSMA (extending to both hemispheres; peak at x, y, z = −4, 8, 58; *Z*_max_ = 3.41), left putamen (peak at x, y, z = −28, 8, −6; *Z*_max_ = 3.50), and right putamen (peak at x, y, z = 34, 0, 4; *Z*_max_ = 3.05), which gradually emerged during the first 20 minutes after L‐dopa intake.

The increased activity was only found in the No‐Go condition, where patients had to actively suppress a motor response. By contrast, left or right button presses were not associated with any between‐group differences in changes of task‐related activity. ASL measurements did not demonstrate significant differences in regional perfusion in bilateral putamen and preSMA between the LID and No‐LID groups, indicating that the observed changes in neural activity were not induced by perfusion differences.

The L‐dopa–induced linear increase of No‐Go activity in preSMA was a strong predictor of LID severity (*R*^2^ = 0.70, adjusted *R*^2^ = 0.67, β = 0.84, *p* < 0.001), explaining ∼70% of the variance of individual dyskinesia scores (see Fig 3B). The L‐dopa–induced increase in preSMA activation did not predict differences in disease severity as indexed by UPDRS‐III‐scores (*p* = 0.83; see Fig 3C). In the putamen, L‐dopa–induced linear increase in No‐Go activity did not predict individual dyskinesia scores or UPDRS‐III scores (all *p* > 0.25).

SVM‐based classification revealed that the abnormal L‐dopa–induced response in preSMA and putamen during No‐Go trials significantly predicted whether patients had a diagnosis of LID (accuracy = 80.8%, sensitivity = 69.2%, specificity = 92.3%, area under the curve [AUC] = 0.87, *p* < 0.001; see Fig 3D). Interestingly, all 3 LID patients who did not develop dyskinesias during the 3 post–L‐dopa fMRI scans were labeled as No‐LID patients by the classifier. Considering only LID patients who developed dyskinesias during the fMRI experiment yielded a sensitivity of 90% and a specificity of 92.3% (AUC = 0.96, *p* < 0.001; see Fig 3E), corresponding to 1 false‐positive and 1 false‐negative prediction.

### Head Movements and Physiological Noise

Overall, <0.1% of all fMRI volumes were affected by head movements >1mm. Mean pulse rate (±SD) was 74 ± 12/min and mean respiration frequency was 16 ± 4/min. Separate ANOVAs yielded no differences between groups and sessions with respect to head movements (ie, relative shifts >1mm between consecutive scans), pulse rate, or respiration frequency.

## Discussion

Using the BOLD signal as index of regional neural activity, we show for the first time that exposure to L‐dopa leads to an excessive increase in activation of the putamen and preSMA in PD patients with peak‐of‐dose dyskinesias. Hyperactivity of the putamen and pre‐SMA in the predyskinesia period was only observed in the behavioral context of response inhibition and was highly predictive of emergence and day‐to‐day severity of dyskinesias.

Within the basal ganglia, excessive activation in PD patients with dyskinesias was restricted to the bilateral putamen. This part of the basal ganglia is most strongly affected by dopaminergic denervation in PD^28^ and is thought to play a central role in the pathophysiology of dyskinesias.^23^ Hypersensitivity of striatal medium spiny neurons to pulsatile dopamine receptor stimulation during task‐related corticostriatal activation of glutamate receptors has been identified as a key alteration in animal models of LID.^8^ Such a mechanism is in good agreement with our observation that the putamen shows an abnormal response to dopamine after prolonged dopaminergic withdrawal only in LID patients, but not in patients without dyskinesias. Furthermore, our findings are in accordance with previous raclopride positron emission tomography studies revealing that patients with dyskinesias display larger increases in striatal synaptic dopamine levels after L‐dopa administration.^29^

In the present study, putaminal hyperactivity emerged rapidly within the first 20 minutes after L‐dopa intake. This predyskinesia response indicates that abnormal dopaminergic stimulation is already present before the threshold for dyskinesias has been reached and demonstrates that excessive activity is not simply a consequence of involuntary movements, which could be the case when comparing a dyskinetic with a nondyskinetic state.^12^

In the LID group, the putamen only became overactive in a context where subjects had to block automatic response tendencies (No‐Go). Current models postulate that the basal ganglia play a key role in motor control.^32^ The neural architecture of loops connecting the cortex and basal ganglia allow rapid inhibition and release of motor programs via the direct, indirect, and hyperdirect pathways.^33^ Importantly, these pathways are differentially modulated by dopamine. Striatal dopamine release induces movement‐facilitating Go feedback via D1‐type dopaminergic receptors and inhibitory No‐Go feedback via D2‐type receptors.^23^ In PD, decreased dopamine release is thought to impair movement facilitation, resulting in bradykinesia. Conversely, the observed overactivity of the putamen in a No‐Go context in PD patients with dyskinesias might reflect an unphysiological facilitation or impaired inhibition of motor programs resulting in aberrant activity in interconnected cortical areas.

At the cortical level, the preSMA was the only area showing increased responsiveness to L‐dopa in the LID group. Moreover, the L‐dopa–induced increase in No‐Go activity in preSMA, but not in putamen, predicted individual severity of dyskinesias. Interestingly, activation of preSMA has been linked to internally generated movements and the intention to act,^35^ including the generation of involuntary actions.^37^ The connectivity of this region to motor regions is abnormal in PD^38^ and modulated by dopamine.^39^ Furthermore, preSMA is involved in decreasing motor threshold during the speed–accuracy tradeoff.^40^ The observed overactivity of preSMA might therefore constitute an aberrant striatal feedback signal that causes an abnormal induction of internally generated movements and hereby contributes to the emergence of dyskinesias.

Previous studies using transcranial magnetic stimulation (TMS) have shown that interrupting function of the caudal SMA alleviates dyskinesias, but only for a limited duration.^41^ The present results raise the question whether the results of TMS might be better if TMS directly targeted the more rostrally located preSMA.

In conclusion, we used a novel fMRI approach to assess the dynamic response of neural regions to a standard dose of fast‐acting soluble L‐dopa. We demonstrate that a rapidly emerging hypersensitivity of putamen and preSMA in the context of movement suppression can be used to predict the onset and severity of dyskinesias in individual PD patients.

## Authorship

D.M.H., M.S.C., K.H.M., A.L., and H.R.S. designed the experiment. D.M.H. and A.L. recruited the participants. D.M.H., B.N.H., and A.L. acquired the data. D.M.H., B.N.H., K.H.M., J.B.R., and H.R.S. analyzed the data. D.M.H., B.N.H., M.S.C., K.H.M., J.B.R., A.L., and H.R.S. wrote the article.

## Potential Conflicts of Interest

A.L.: speaking fees, Lundbeck Pharma, Medtronic; advisory board, UCB; nonfinancial support, Abbott, UCB, Medtronic. H.R.S.: advisory board, Lundbeck; editor, *NeuroImage*; royalties, Springer; speaking fees, Biogen Idec, Genzyme; travel support, MagVenture; grants, Lundbeck Foundation, John and Birthe Meyer Foundation, Fri Forskningsråd for Sundhed og Sygdom, Region Hovedsteden Forskningsfond, Fri Forskningsråd for Samfund og Erhverv Ludiomaniprogrammet.
